# Improved Clinical Outcome of COVID-19 in Hematologic Malignancy Patients Receiving a Fourth Dose of Anti-SARS-CoV-2 Vaccine: An EPICOVIDEHA Report

**DOI:** 10.1097/HS9.0000000000000789

**Published:** 2022-10-25

**Authors:** Jon Salmanton-García, Francesco Marchesi, Andreas Glenthøj, Yavuz M. Bilgin, Jens van Praet, Julio Dávila-Valls, Sonia Martín-Pérez, Jorge Labrador, Jaap van Doesum, Iker Falces-Romero, Francesca Farina, Martin Schönlein, Mathilde Chanut, Verena Petzer, Ildefonso Espigado, Michelina Dargenio, Avinash Aujayeb, Uluhan Sili, Laura Serrano, László Imre Pinczés, Nick de Jonge, Andrés Soto-Silva, Caterina Buquicchio, Lucia Prezioso, Monia Marchetti, Stef Meers, Alessandro Busca, Paolo Corradini, Martin Hoenigl, Philipp Koehler, Laman Rahimli, Gökçe Melis Çolak, Elena Arellano, Dominik Wolf, Stefanie Gräfe, Emanuele Ammatuna, Caroline Berg Venemyr, Oliver A. Cornely, Livio Pagano

**Affiliations:** 1 University of Cologne, Faculty of Medicine and University Hospital Cologne, Translational Research, Cologne Excellence Cluster on Cellular Stress Responses in Aging-Associated Diseases (CECAD), Germany; 2 University of Cologne, Faculty of Medicine and University Hospital Cologne, Department I of Internal Medicine, Center for Integrated Oncology Aachen Bonn Cologne Duesseldorf (CIO ABCD) and Excellence Center for Medical Mycology (ECMM), Germany; 3 Hematology and Stem Cell Transplant Unit, IRCCS Regina Elena National Cancer Institute, Rome, Italy; 4 Department of Hematology, Copenhagen University Hospital—Rigshospitalet, Denmark; 5 Department of Internal Medicine, ADRZ, Goes, the Netherlands; 6 Department of Nephrology and Infectious diseases, AZ Sint-Jan Brugge-Oostende AV, Belgium; 7 Hospital Nuestra Señora de Sonsoles, Ávila, Spain; 8 Department of Hematology, Research Unit, Hospital Universitario de Burgos, Spain; 9 University Medical Center Groningen, the Netherlands; 10 La Paz University Hospital, Madrid, Spain; 11 IRCCS Ospedale San Raffaele, Milan, Italy; 12 Department of Oncology, Hematology and Bone Marrow Transplantation with Section of Pneumology, University Medical Center Hamburg-Eppendorf, Germany; 13 Centre Hospitalier de Versailles, Le Chesnay, Université Paris-Saclay, UVSQ, Inserm, Équipe “Exposome et Hérédité”, CESP, Villejuif, France; 14 Department of Hematology and Oncology, Comprehensive Cancer Center Innsbruck (CCCI), Medical University of Innsbruck (MUI), Innsbruck, Austria; 15 Department of Hematology, University Hospital Virgen Macarena–University Hospital Virgen del Rocío, Instituto de Biomedicina de Sevilla (IBIS/ CSIC), Universidad de Sevilla (Departamento de Medicina), Spain; 16 Hematology and Stem Cell Transplant Unit, Vito Fazzi, Lecce, Italy; 17 Northumbria Healthcare, Newcastle, United Kingdom; 18 Marmara University, Istanbul, Turkey; 19 Hospital Universitario de Cabueñes, Gijón, Spain; 20 Division of Hematology, Department of Internal Medicine, University of Debrecen, Hungary; 21 Amsterdam UMC, Location VUmc, Amsterdam, the Netherlands; 22 Faculty of Medicine, University of Chile. Infectious Diseases Unity, Salvador Hospital of Santiago, Santiago de Chile, Chile; 23 Ematologia con Trapianto, Ospedale Dimiccoli Barletta, Italy; 24 Hospital University of Parma - Hematology and Bone Marrow Unit, Parma, Italy; 25 Azienda Ospedaliera Nazionale SS. Antonio e Biagio e Cesare Arrigo, Alessandria, Italy; 26 AZ KLINA, Brasschaat, Belgium; 27 Stem Cell Transplant Center, AOU Citta’ della Salute e della Scienza, Turin, Italy; 28 University of Milan and Fondazione IRCCS Istituto Nazionale dei Tumori, Milan, Italy; 29 Division of Infectious Diseases and Global Public Health, Department of Medicine, Clinical and Translational Fungal-Working Group, University of California San Diego, CA, USA; 30 Division of Infectious Diseases, Department of Internal Medicine, Medical University of Graz, Austria; 31 Marmara University, Istanbul, Turkey; 32 Department of Hematology, University Hospital Virgen Macarena, Seville, Spain; 33 Department of Hematology and Oncology, Comprehensive Cancer Center Innsbruck (CCCI), Medical University of Innsbruck (MUI), Austria; 34 Universitätsklinikum Hamburg Eppendorf, Germany; 35 Clinical Trials Centre Cologne (ZKS Köln), Center for Molecular Medicine Cologne (CMMC), University of Cologne, Faculty of Medicine and University Hospital Cologne, Germany; 36 German Centre for Infection Research (DZIF), Partner Site Bonn-Cologne, Cologne, Germany; 37 Hematology Unit, Fondazione Policlinico Universitario Agostino Gemelli - IRCCS, Rome, Italy; 38 Hematology Unit, Università Cattolica del Sacro Cuore, Rome, Italy

Severe acute respiratory syndrome coronavirus 2 (SARS-CoV-2) causes life-threatening COVID-19 in hematologic malignancy (HM) patients, associated with high morbidity and mortality in this particularly vulnerable population.^1^ After more than 2 years since the beginning of the COVID-19 pandemic, several prophylactic and therapeutic strategies have been developed against SARS-CoV-2, including targeted antivirals, monoclonal antibodies and vaccines, leading to improved prognosis in HM patients.^2^ Our preliminary data in vaccinated HM patients with COVID-19 reported a significant reduction in disease severity and mortality rate, however, still exceeding the mortality rate in the overall population.^3^

As a consequence of breakthrough SARS-CoV-2 infections in fully vaccinated patients who had received a booster or third dose,^4^ a second booster or fourth dose was approved in Europe at the beginning of 2022. Despite the absence of strong clinical evidence, target populations were defined as advanced age or high-risk individuals (eg, patients suffering from HM) who had received a third dose at least 4 months before.^5–7^ However, the lack of consolidated data has resulted in a general skepticism on anti-SARS-CoV-2 fourth dose efficacy among both, the public and the scientific community. As a consequence, so far even among HM patients, only a very few individuals received a fourth dose, despite several data supports the low immunogenicity of anti-SARS-CoV-2 vaccine in this patient population, particularly in those treated with immunosuppressive agents.^8^

Here, we report the clinical presentation and outcome of COVID-19 occurring in a cohort of HM patients who had previously received a fourth anti-SARS-CoV-2 vaccine dose registered in the EPICOVIDEHA registry.

EPICOVIDEHA (clinicaltrials.gov: NCT04733729) is an open online registry gathering clinical data from COVID-19 patients with baseline HM, hosted at www.clinicalsurveys.net (EFS Summer 2021, TIVIAN, Cologne, Germany). The survey is an initiative of the European Hematology Association—Infectious Diseases Working Party (EHA-IDWP), with a central approval from the Institutional Review Board and Ethics Committee of Fondazione Policlinico Universitario A. Gemelli—IRCCS—Università Cattolica del Sacro Cuore, Rome, Italy (Study ID: 3226). When applicable, the respective local ethics committee of each participating institution approved the project. Detailed EPICOVIDEHA methods have been described elsewhere.^9^ Each documented patient was reviewed and validated by infectious diseases and hematology experts from the coordination team. Inclusion criteria were (1) active HM within the last five years before COVID-19 diagnosis, (2) patients ≥18 years old, (3) laboratory-based diagnosis of SARS-CoV-2 infection, and (4) reception of a fourth anti-SARS-CoV-2 dose before COVID-19 diagnosis. COVID-19 severity was graded according to international standards, as previously described.^1,9^ Between April 2022 and June 2022, up to 160 contributors could provide data from more than 40 countries. Categorical variables have been summarized in frequencies and percentages, whereas continuous variables in median and interquartile range (IQR). No further inferential analyses have been performed due to the limited sample size.

As of August 2022, 7302 HM patients with SARS-CoV-2 infection have been registered in the EPICOVIDEHA registry. Of these, 102 (1.4%) were diagnosed with COVID-19 after having received a fourth vaccine dose (Table 1); these patients were registered from 23 sites located in 12 European countries. In the large majority of cases (94%) this was the first occurrence of COVID-19, whereas only 6 patients had been already affected by COVID-19 in the past. Most of those (66/102, 64.7%) were male; median age was 69 years (IQR 62–75) comprising only 9 patients (8.8%) below the age of 50 years.

**Table 1:** Baseline Characteristics of the Patients and Clinical Outcome

	n	%
Sex		
Female/male	36/66	35.3/64.7
Median age (IQR)	69 (62–75)	
Baseline malignancy		
Non-Hodgkin lymphoma	38	37.3
Plasma cell disorders	26	25.5
Chronic lymphoid leukemia	18	17.6
Acute myeloid leukemia	8	7.8
Myelodisplastic syndrome	4	3.9
Acute lymphoid leukemia	3	2.9
Hodgkin lymphoma	2	2.0
Chronic myeloid leukemia	1	1.0
Myelofibrosis	1	1.0
Polycythemia vera	1	1.0
Status malignancy at COVID-19 diagnosis		
Controlled	63	61.8
Active	38	37.3
Unknown	1	1.0
Last antineoplastic treatment before COVID-19		
Immunochemotherapy	58	56.9
Targeted therapy	14	13.7
AlloHSCT	8	7.8
AutoHSCT	3	2.9
Conventional chemotherapy	3	2.9
Demethylating agents	3	2.9
Supportive measures	3	2.9
CAR-T	2	2.0
Immunotherapy	1	1.0
No treatment	7	6.9
Last vaccine before COVID-19		
Median days before COVID-19 diagnosis (IQR)	42 (20–64)	
mRNA	101	99.0
Inactivated	1	1.0
SARS-CoV-2 variant		
*Delta*	1	1.0
*Omicron*	43	42.2
BA 1	5	4.9
BA 2	18	17.5
BA 4/5	4	3.9
Unknown	16	15.7
Not tested	58	56.9
COVID-19 severity		
Asymptomatic	10	6.9
Mild infection	49	48.0
Severe infection	39	38.2
Critical infection	4	3.9
Neutrophils at COVID-19 diagnosis		
≥1000 cells/mm^3^	68	66.7
Lymphocytes at COVID-19 diagnosis		
≥500 cells/mm^3^	60	58.8
Stay during COVID-19 episode		
Hospital	39	38.2
Length of stay	10 (7–20)	
Due to COVID-19	31	30.4
Oxygen administration	22	21.6
Home	63	61.8
COVID-19 treatment		
No specific treatment reported	46	45.1
Antivirals ± convalescent plasma	20	19.6
Monoclonal antibodies ± convalescent plasma	14	13.7
Antivirals + monoclonal antibodies ± convalescent plasma	12	11.8
Corticosteroids	10	9.8
Outcome		
Median observation time since COVID-19 diagnosis (IQR)	54 (27–82)	
Alive Dead	98 4	96.1 3.9

alloHSCT = allogeneic hematopoietic stem cell transplant; autoHSCT = autologous hematopoietic stem cell transplant; CAR-T = chimeric antigen receptor T-cells; IQR = interquartile range.

Almost 50% of the patients had an underlying chronic cardiopathy (50/102, 49.0%), 15.7% (16/102) suffered from diabetes mellitus, and 12.7 % (13/102) had a chronic pulmonary diseases or smoking history, each. Thirty patients (30/102, 29.4%) did not present any baseline underlying conditions besides the HM. Lymphoproliferative malignancies were prevalent (86/102, 84.3%), especially non-Hodgkin lymphoma (38/102, 37.3%), plasma cell disorders (26/102, 25.5%), and chronic lymphocytic leukemia (18/102, 25.517.6%).

Eight patients had received an allogeneic stem cell transplantation before COVID-19 diagnosis, 6 of which with baseline acute myeloid leukemia (AML)/high-risk myelodysplastic syndrome. Most patients had a controlled HM at the time of COVID-19 diagnosis (63/102, 61.8%), whereas the remaining suffered from active disease (38/102, 37.3%). More than half of the patients (58/102, 56.9%) received immunochemotherapy as the most recent antineoplastic treatment before COVID-19, the majority of which (42/58, 72.4%) received therapy within 3 months before COVID-19 diagnosis. At the time of COVID-19 diagnosis, neutrophil counts were higher than 1000/mm³ in 68 patients (66.7%) and lymphocyte counts were above 500/mm³ in 58.8% (60/102).

Patients received the fourth vaccine dose at a median of 42 days (IQR 20–62) before COVID-19 diagnosis, almost exclusively mRNA based (101/102, 99.0%). Genotyping of the VOC was performed in 43.2% (44/102) of patients and identified the *Omicron* variants in all samples but one; the most frequently reported sub-variants was BA.2 (Table 1). COVID-19 remained asymptomatic or mild in almost all cases (59/102, 57.8%), whereas 39 cases were defined as severe (38.2%); only 4 cases of critical infection (3.9%) requiring intensive care. Hospital admission rate was 38.2% with a median hospital stay of 10 days (IQR 7–21). Only 56 patients (54.9%) received a specific treatment for SARS-CoV-2, with almost all of them (26/56, 46.4%) receiving monoclonal antibodies. Patients were followed up for a median of 54 days (IQR 27-83) and only four died (3.9%); in two cases the death was attributable to COVID-19, whereas in the others 2, it was due to both COVID-19 and HM. All patients were older than 70 years, 3 (75%) had comorbidities, were affected by relapsed/refractory lymphoproliferative malignancies and were receiving immunochemotherapy at the time of COVID-19. In both cases genotyping revealed the *Omicron* BA.2 variants.

To the best of our knowledge, no studies have been previously published regarding clinical presentation and outcome of COVID-19 in HM patients who had received the second anti-SARS-CoV-2 vaccine booster (fourth dose). From April to August 2022, we collected 102 cases in EPICOVIDEHA who had breakthrough COVID-19 after 4 vaccine doses. Viral genotyping was available in only 43.2% of patients but showed consistently *Omicron* VOC. Considering the trajectory of the pandemic, we assume that all cases registered most likely are attributable to *Omicron*. The clinical presentation was different from reports in the prevaccination era and in our previous study on breakthrough infections in HM patients who had received two vaccine doses. After the fourth vaccination doses, the rate of severe/critical cases observed in patient with COVID-19 was significantly lower when compared to our previous reports (63.8%/70% versus 42.1%; *P* < 0.001).^1,3^ Accordingly, we observed a very low overall mortality rate, since only 4 deaths were reported (3.9%) in patients receiving four vaccination doses. The largest number of patients presented with mild symptoms (48.0%) and most of them were easily managed at home.

About half of patients received specific treatments, mostly monoclonal antibodies and only a few patients received antiviral compounds. These data are interesting and even more impressive if compared with our previously published experience on a large cohort of HM patients with *Omicron* VOC infection, in which we found an hospital admission rate (52%) and overall mortality rate (8.6%). Of note, in this last study, at least 1 vaccine dose had been administered to 83.1% of patients, but only a very few of them (1%) received a fourth dose.^10^

In accordance with several reports, most patients were affected by lymphoproliferative malignancies, and baseline demographic and clinical characteristics were similar to those observed in our previous experiences and in the prevaccination era.^1,2^ Moreover, the majority of our patients were treated with immunochemotherapy or targeted treatments, both of them well recognized risk-factors for poor response to anti-SARS-CoV-2 vaccines.^11^ Interestingly, in this series we found only 15 patients affected by myeloproliferative malignancies (14.7%); this is consistent with the putative higher immunogenicity of anti-SARS-CoV-2 vaccine in patients affected by myeloid malignancies.^12^ Consistently, 4 of 8 AML patients reported in our cohort had previously received allogeneic stem cell transplant, which probably made them less capable of mounting an adequate humoral immune response, as observed in lymphoproliferative malignancies.^8^

Unfortunately, we had too little data about serological responses upon the fourth vaccine dose, since a post-vaccine evaluation of antispike immunoglobulin G was not routinely assessed at the enrolling sites; this is a weakness of our study limiting the understanding of the real efficacy of the fourth vaccine dose, at least in terms of its capability to induce humoral immune responses. Moreover, from our data it’s difficult to definitely establish whether the prognosis improvement is truly related to the 2 extra booster vaccine doses. The success of vaccination strategies is likely a major but not the only factor; a better COVID-19 management and the less severity of newer variants may have played a significant role as well. Previous reports suggest that COVID-19 management (eg, steroids, antivirals, monoclonal antibodies) have also impacted outcomes.

In conclusion, with the caution derived from the limited patient number and the intrinsic nature of this study, our data show favorable clinical presentation and outcome of breakthrough SARS-CoV-2 infections in HM who previously received a fourth dose of anti-SARS-CoV-2 vaccine. These data suggest that a second vaccine booster may be of particular importance to protect this particularly vulnerable patient population from severe or potentially life-threatening COVID-19.

## ACKNOWLEDGMENTS

The authors thank all contributors for their utmost contributions and support to the project during a pandemic situation.

## AUTHOR CONTRIBUTIONS

LP served as the principal investigator. JS-G, FM, and LP contributed to study design, study supervision, and data interpretation and wrote the paper. JS-G, FM, and LP did the statistical plan, analysis and interpreted the data. All the authors recruited participants and collected and interpreted data. All authors contributed to manuscript writing and review of the manuscript. All authors agreed to be accountable for all aspects of the work in ensuring that questions related to the accuracy or integrity of any part of the work are appropriately investigated and resolved.

## DISCLOSURES

PC is a HemaSphere Editor. The authors have no conflicts of interest to disclose.

## SOURCES OF FUNDING

EPICOVIDEHA has received funds from Optics COMMITTM (COVID-19 Unmet Medical Needs and Associated Research Extension) COVID-19 RFP program by GILEAD Science, United States (Project 2020-8223). The funder of the study had no role in study design, data analysis, interpretation, or writing of the report. All authors had full access to the data and had final responsibility for the decision to submit for publication.
